# Leaf traits drive plant diversity effects on litter decomposition and FPOM production in streams

**DOI:** 10.1371/journal.pone.0198243

**Published:** 2018-05-29

**Authors:** Naiara López-Rojo, Aingeru Martínez, Javier Pérez, Ana Basaguren, Jesús Pozo, Luz Boyero

**Affiliations:** 1 Department of Plant Biology and Ecology, Laboratory of Stream Ecology, University of the Basque Country (UPV/EHU), Bilbao, Spain; 2 IKERBASQUE, Basque Foundation for Science, Bilbao, Spain; 3 College of Science and Engineering, James Cook University, Townsville, Australia; Estacion Experimental de Zonas Aridas, SPAIN

## Abstract

Biodiversity loss in riparian forests has the potential to alter rates of leaf litter decomposition in stream ecosystems. However, studies have reported the full range of positive, negative and no effects of plant diversity loss on decomposition, and there is currently no explanation for such inconsistent results. Furthermore, it is uncertain whether plant diversity loss affects other ecological processes related to decomposition, such as fine particulate organic matter production or detritivore growth, which precludes a thorough understanding of how detrital stream food webs are impacted by plant diversity loss. We used a microcosm experiment to examine the effects of plant diversity loss on litter decomposition, fine particulate organic matter production, and growth of a dominant leaf-shredding detritivore, using litter mixtures varying in species composition. We hypothesized that plant diversity loss would decrease the rates of all studied processes, but such effects would depend on the leaf traits present in litter mixtures (both their average values and their variability). Our findings partly supported our hypotheses, showing that plant diversity loss had a consistently negative effect on litter decomposition and fine particulate organic matter production (but not on detritivore growth) across litter mixtures, which was mediated by detritivores. Importantly, the magnitude of the diversity effect and the relative importance of different mechanisms underlying this effect (i.e., complementarity vs. selection) varied depending on the species composition of litter mixtures, mainly because of differences in litter nutritional quality and trait variability. Complementarity was prevalent but varied in size, with positive selection effects also occurring in some mixtures. Our results support the notion that loss of riparian plant species is detrimental to key stream ecosystem processes that drive detrital food webs, but that the magnitude of such effects largely depends on the the order of species loss.

## Introduction

Human activities are altering freshwater ecosystems worlwide, with significant impacts on biological communities and ecosystem processes [1]. The removal of riparian vegetation, which often occurs in association with agriculture, deforestation or afforestation with exotic species, can affect key stream ecosystem processes such as leaf litter decomposition [2]. This is particularly evident in forested streams, where inputs of dead organic matter from the surrounding vegetation constitute the basis of a detrital food web [3]. Leaf litter is used by microbial decomposers and by invertebrate leaf-shredding detritivores, which incorporate the plant material into animal biomass [4]. Microbial and detritivore activity, together with mechanical fragmentation, also lead to the production of fine particulate organic matter (FPOM), which provides an important resource for other consumers such as collector-gatherers and filter-feeders [5, 6], ultimately supporting invertebrate and vertebrate predators [3]. Understanding how these key processes are affected by biodiversity loss is important to predict alterations in stream ecosystem functioning [7], given the current high rates of extinction [8].

The consequences of riparian plant diversity loss for decomposition in streams have been extensively studied. However, this relationship is still unclear because studies have found the full range of positive, negative and no effects of plant diversity loss on decomposition [9]. Furthermore, we know little about the potential effects of plant diversity loss on processes associated with decomposition, such as detritivore growth and FPOM production, which are likely to have top-down and bottom-up effects for the detrital food web, respectively [6, 10]. Thus, our understanding of how detrital stream food webs are impacted by the loss of riparian plant diversity is limited.

We examined the effects of plant diversity loss on the rates of litter decomposition, FPOM production and detritivore growth in streams, using a microcosm experiment with four riparian plant species (*Alnus glutinosa* L. Gaertner, *Corylus avellana* L., *Quercus robur* L. and *Ilex aquifolium* L.; hereafter *Alnus*, *Corylus*, *Quercus* and *Ilex*) and a detritivore species (*Sericostoma pyrenaicum* Pictet), all of which are common in our study area. Firstly, we hypothesized that plant diversity loss would lead to a general decrease in litter decomposition [11], but the magnitude of such effect would vary depending on the leaf traits present initially in litter mixtures. Thus, we examined the effects of losing plant diversity on decomposition not only from the 4-species mixture composed of *Alnus* (A), *Corylus* (C), *Quercus* (Q) and *Ilex* (I), but also from the four different possible 3-species mixtures (i.e., ACQ, ACI, AQI and CQI), which differed in their average leaf traits and variability.

Secondly, we hypothesized that the effect of plant diversity on decomposition would be due to a combination of positive complementarity (effects resulting from synergistic interactions) and positive selection (effects due to the presence of a species with particularly high decomposition rates) [12], but the relative importance of both mechanisms would vary depending on the species present in the mixture. Specifically, we expected that selection effects would become more important in litter mixtures containing *Alnus*, because litter from this species decomposes faster than many other species and is often preferred by detritivores [13, 14]. Finally, we hypothesized that effects of plant diversity loss on FPOM production and detritivore growth would be similar to those on decomposition, as these threee processes are intimately related, although our experimental design did not allow exploring the relative role of complementarity and selection effects on FPOM production and detritivore growth because the contribution of different plant species to these proceses cannot be distinguished in polycultures.

## Materials and methods

We selected four plant species that were present in the riparian forest and belonged to different plant functional types: a nitrogen (N) fixer (*Alnus*), a deciduous fast decomposer (*Corylus*), a deciduous slow decomposer (*Quercus*) and an evergreen species (*Ilex*). Although *Ilex* was less abundant than the other species, its relative contribution to stream leaf litter was considerable in seasons other than autumn (pers. obs.). The species differed in several leaf traits (Table 1): N and phosphorus (P) concentrations [% dry mass (DM)]; specific leaf area [SLA; ratio of disc area (mm^2^) to leaf DM (mg)]; leaf toughness [measured as the pressure required to pierce the leave tissue using a steel rod (kPa)]; and ash concentration [(% DM remaining after high-temperature combustion), which is a surrogate of leaf defense [15]]. We collected leaves from the Agüera stream catchment in northern Spain (43.20^o^N, 3.26^o^W) in the autumn of 2015. Leaves of deciduous species were collected from the forest floor immediately after natural abscission. For the evergreen *Ilex*, as there is no peak of abscission that allows the collection of leaves at one time, we followed Handa et al.'s procedure [12] and collected branches, which were stored in the laboratory until the leaves were dry. Leaf discs of 12-mm diameter were cut with a cork borer avoiding the basal midrib, air dried, and weighed to the nearest 0.01mg using a precision balance in groups of 12, 16, 24 or 48 discs.

**Table 1:** Mean (± SE) of nitrogen (N) and phosphorus (P) concentrations (% DM), specific leaf area (SLA; mm^2^ mg^-1^), leaf toughness (kPa) and ash concentration (% DM) for each plant species (based on measurements of five replicates) and litter mixture, and trait distance in each litter mixture based on cluster analysis. Different letters indicate significant differences (*p* < 0.05) across single species and 3-spps litter mixtures, on the basis of linear models followed by pairwise multiple comparisons.

	N	P	SLA	Toughness	Ash	Trait distance
**Plant species**						
*Alnus glutinosa* (A)	3.30 ± 0.16^a^	0.084 ± 0.004^a^	13.28 ± 0.34^b^	1397 ± 75^c^	5.64 ± 0.31^a^	
*Corylus avellana* (C)	1.48 ± 0.03^b^	0.053 ± 0.001^c^	21.90 ± 1.04^a^	1016 ± 44^c^	5.66 ± 0.18^a^	
*Quercus robur (Q)*	1.23 ± 0.07^b^	0.041 ± 0.004^d^	11.50 ± 0.15^b^	2793 ± 158^b^	4.68 ± 0.19^b^	
*Ilex aquifolium* (I)	1.58 ± 0.05^b^ *	0.068 ± 0.002^b^	6.57 ± 0.26^c^	7715 ± 100^a^	3.93 ± 0.05^b^	
**Litter mixtures**						
ACQI	1.80 ± 0.007	0.062 ± 0.0002	13.31 ± 1.29	4751 ± 43.34	4.64 ± 0.01	3.43 ± 0.001
ACQ	2.09 ± 0.01^a^	0.060 ± 0.0002 ^c^	15.56 ± 1.26 ^a^	2263 ± 9.31 ^c^	5.26 ± 0.01 ^a^	3.30 ± 0.008^c^
ACI	2.02 ± 0.02^b^	0.069 ± 0.0002 ^a^	13.91 ± 1.71 ^b^	5246 ± 84.35 ^b^	4.66 ± 0.02 ^b^	3.69± 0.005^a^
AQI	1.88 ± 0.005^c^	0.064 ± 0.002 ^b^	10.44 ± 0.77 ^c^	5059 ± 69.76 ^b^	4.49 ± 0.01^c^	3.56± 0.003^b^
CQI	1.46 ± 0.002^d^	0.058 ± 0.002 ^d^	13.32 ± 1.74 ^b^	5521 ± 53.48 ^a^	4.42 ± 0.01^d^	3.27 ± 0.005^c^

*N concentration of *Ilex aquifolium* leaves used in the experiment (which were collected from branches) did not significantly differ from a sample of senescent leaves collected from the ground (1.62 ± 0.13% DM; t-test, t = -0.28, df = 2.54, p = 0.601).

The experiment was carried out in May 2016 in 150 microcosms placed in a temperature-controlled room kept at 10°C (in order to mimic natural conditions in this region and minimize evaporation), with constant aeration and a light:dark regime of 12:12 h. The microcosms consisted of 500-mL glass jars containing 400 mL of stream water (soluble reactive P: 10.0± 0.9 μg P L^-1^; dissolved inorganic N: 453.6 ± 30.4 μg N L^-1^; n = 4) filtered through 100-μm mesh, which allowed the entrance of microorganisms. Each microcosm received 48 leaf discs that belonged to 1 plant species (monocultures) or to 2, 3 or 4 species (i.e., litter mixtures containing 24, 16 or 12 discs per species, respectively), with 10 microcosms per treatment. Leaf discs were incubated for 48 h to allow the leaching of soluble compounds and initial microbial conditioning, and after that the water was replaced with filtered (100 μm) stream water, and detritivores were added to half of the microcosms; the other half remained without detritivores, which allowed separating effects mediated by detritivores and microorganisms.

Microcosms with detritivores received 3 larvae of the caddisfly *Sericostoma pyrenaicum* (hereafter *Sericostoma*), which had been starved for 48 h prior to the experiment. Initial detritivore dry mass (DM) was estimated from a case length (CL) (mm) /DM (mg) relationship, using 45 additional larvae collected simultaneously as experimental individuals and with a similar case length range (DM = 0.0043 × CL^2.8041^; r^2^ = 0.79). Case length was measured under a binocular microscope with an accuracy of 0.5 mm, and then individuals were uncased, freeze-dried and weighed. Detritivore initial biomass per microcosm was on average (± SE) 17.82 ± 0.36 mg.

During the experiment, water was again replaced on days 7 and 14, and the experiment finished on day 21. On each occasion, water from microcosms was firstly filtered through 100-μm mesh to retain the invertebrates and leaf discs and fragments, and water was replaced with filtered (100 μm) stream water. The outgoing water was filtered through preweighed glass fibre filters (Whatman GF/F; pore size: 0.7-μm), and filters were oven-dried (70°C, 72 h), ashed (500°C, 4 h) and weighed (mg) to estimate FPOM production (water entering the microcosms on each occasion had no FPOM, which was determined as explained for outgoing water). On day 21 all the leaf material was separated by species, oven-dried and weighed to determine final DM, and ashed and re-weighed to determine final AFDM. Detritivores from each microcosm were uncased, freeze-dried and weighed to calculate their final DM.

Twenty extra microcosms (5 per species, each containing 48 pre-weighed leaf discs) were used to estimate initial (post-leaching) AFDM of leaf discs, as well as leaf traits (N, P and ash concentrations and SLA). Leaf discs were removed after 48 h, oven-dried (70°C, 72 h), weighed (mg), and SLA was estimated by dividing total leaf disc area (mm^2^) by DM (mg). Discs from each microcosm were then divided into two subsamples; one was ashed (550°C, 4h), and re-weighed to estimate initial AFDM and ash concentration (% DM). The other subsample was used to measure N and P concentrations: N concentration (% DM) was determined using a Perkin Elmer series II CHNS/O elemental analyser, and P concentration (% DM) was measured spectrophotometrically after autoclave-assisted extraction [16]. Additionally, four microcosms were used to leach 25 leaf discs per species for 48 h and to determine leaf toughness using a penetrometer with a 0.79-mm diameter steel rod [17]; for each species we calculated the average for each set of 5 measurements, resulting in 5 final values.

### Data analysis

We quantified litter decomposition through litter mass loss, calculated as the difference between initial and final litter AFDM; initial AFDM was corrected for mass loss due to leaching using the correction factor obtained from extra microcosms (0.753 for *Alnus*, 0.843 for *Corylus*, 0.844 for *Quercus* and 0.767 for *Ilex*). In microcosms with detritivores, we divided litter mass loss by detritivore initial DM in order to remove possible effects due to slight differences in detritivore size across microcosms. FPOM production was calculated as the accumulated FPOM collected in successive water replacements for each microcosm, divided by detritivore initial DM in microcosms with detritivores. Detritivore growth was quantified as the % change in detritivore DM: [(final DM–initial DM) / initial DM] ×100.

We examined the differences in initial leaf traits (N and P concentrations, SLA, toughness and ash concentration) across plant species and across 3-spp litter mixtures with linear models using the *gls* function (generalized least squares) and restricted maximum likelihood (REML) method in the ‘nlme’ R package (version 3.2.5; R Development Core Team 2016), with plant species as a fixed factor, followed by Tukey pairwise multiple comparisons using the glht function of the ‘multcomp’ package [18].

**Fig 1:**
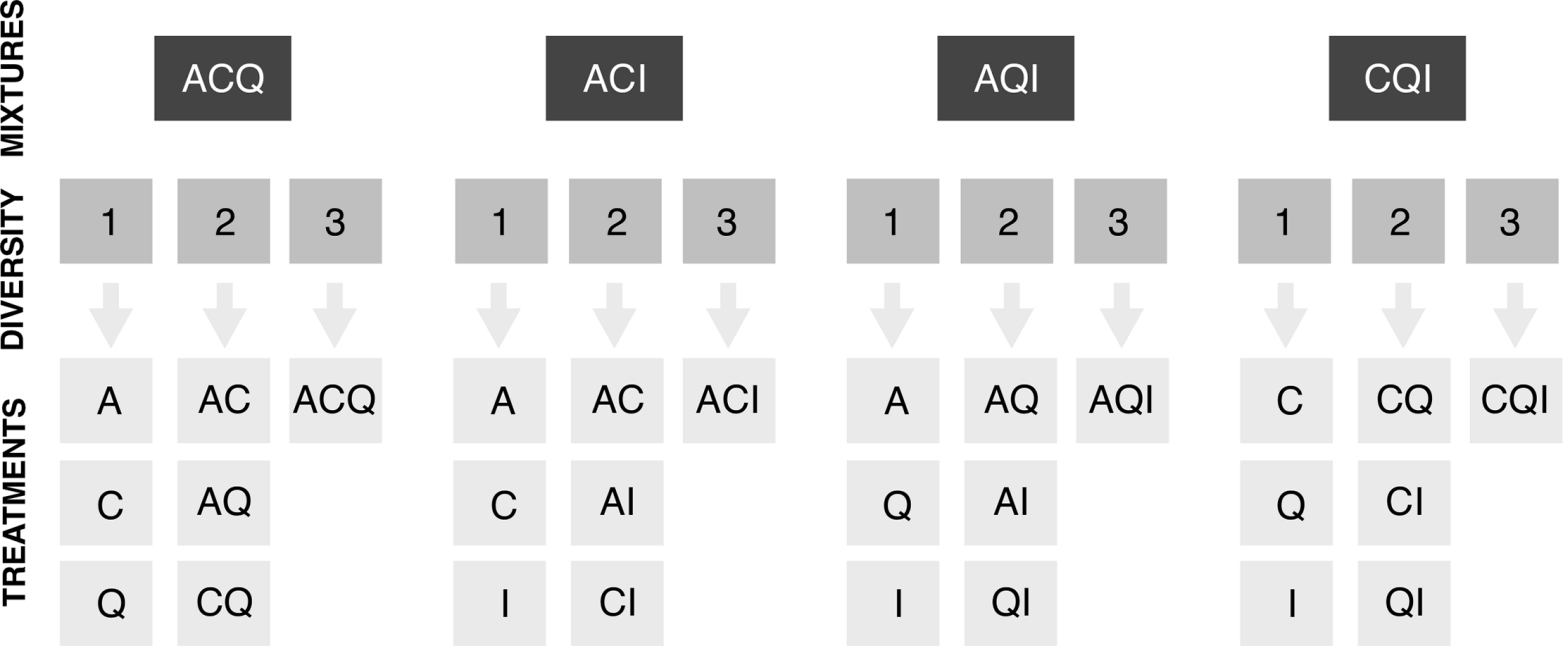
Experimental treatments for each litter mixture and plant diversity level. A: *Alnus glutinosa*; C: *Corylus avellana*; Q: *Quercus robut*; I: *Ilex aquifolium*.

We explored whether litter decomposition, FPOM production and detritivore growth decreased with diversity loss from 4 to 1 species (categorical variable) in the ACQI litter mixture, with linear models followed by pairwise multiple comparisons (as above). For decomposition and FPOM production we did separate analyses for microcosms with and without detritivores. Subsequently, we split the dataset in 4 subsets consisting of 3-species litter mixtures (ACQ, ACI, AQI, CQI; Fig 1) by removing one species each time (e.g., *Ilex* was removed in ACQ). We examined effects of diversity loss from 3 to 1 species in the ACQ, ACI, AQI and CQI litter mixtures using linear models followed by pairwise multiple comparisons (e.g., the ACQ 3-spp mixture was compared with the AC, AQ and CQ 2-spp mixtures and the A, C and Q monocultures). Multi-panel plots for each model showed that the homogeneity of variances assumption was violated, requiring the use of a variance structure that takes these differences into account [VarIdent function of ‘nlme’ R package [19]].

In cases where diversity loss had a significant effect on decomposition, we partitioned the net diversity effect into complementarity and selection effects using the additive partitioning method [20]. The net diversity effect is the difference between observed and expected decomposition (the latter being estimated based on monocultures); the complementarity effect is the average deviation from expected decomposition in a mixture multiplied by mean decomposition in monocultures and by the number of species in the mixture; and the selection effect is the covariance between decomposition of species in monoculture and the average deviation from expected decomposition of species in the mixture, multiplied by the number of species in the mixture [20]. Thus, complementarity results from synergistic or antagonistic interactions, while selection arises when the presence of a particular species with high or low process rates dominates the mixture [12]. Additive partitioning was not applied to FPOM production or detritivore growth because it was not possible to separate the contribution of different plant species to these processes in litter mixtures.

We compared the net diversity, complementarity and selection effects on litter decomposition, and the net diversity effect on FPOM production (calculated as the difference between observed and expected values, as described for litter decomposition), across 3-spp litter mixtures (ACQ, ACI, AQI and CQI) with linear models followed by pairwise multiple comparisons. Further, we explored whether the net diversity, complementarity and selection effects on litter decomposition and the net diversity effect on FPOM production (all continuous variables) were related to average leaf trait values within a mixture (corrected by the DM of each species in the mixture) and their varaibility using linear models; data were log-transformed to comply with linear model assumptions. Leaf trait variability was estimated as the mean distance between species pairs in a mixture; the distance between species pairs was calculated using cluster analysis on standardized data based on all the measured leaf traits using JMP 9.0.1 software (www.jmp.com).

## Results

Plant species differed in all the leaf traits examined: N concentration was higher in *Alnus* than in the other species; P concentration was highest in *Alnus* and lowest in *Quercus*; SLA was highest in *Corylus* and lowest in *Ilex*; toughness was highest in *Ilex* and lowest in *Alnus* and *Corylus*; and ash concentration was higher in *Alnus* and *Corylus* than in the other two species (Table 1). The ACQ mixture had the highest N concentration and SLA and the lowest toughness; P concentration was highest in ACI; ash concentration was highest in ACQ and lowest in CQI; and trait variability was highest in ACI followed by AQI (Table 1).

**Fig 2:**
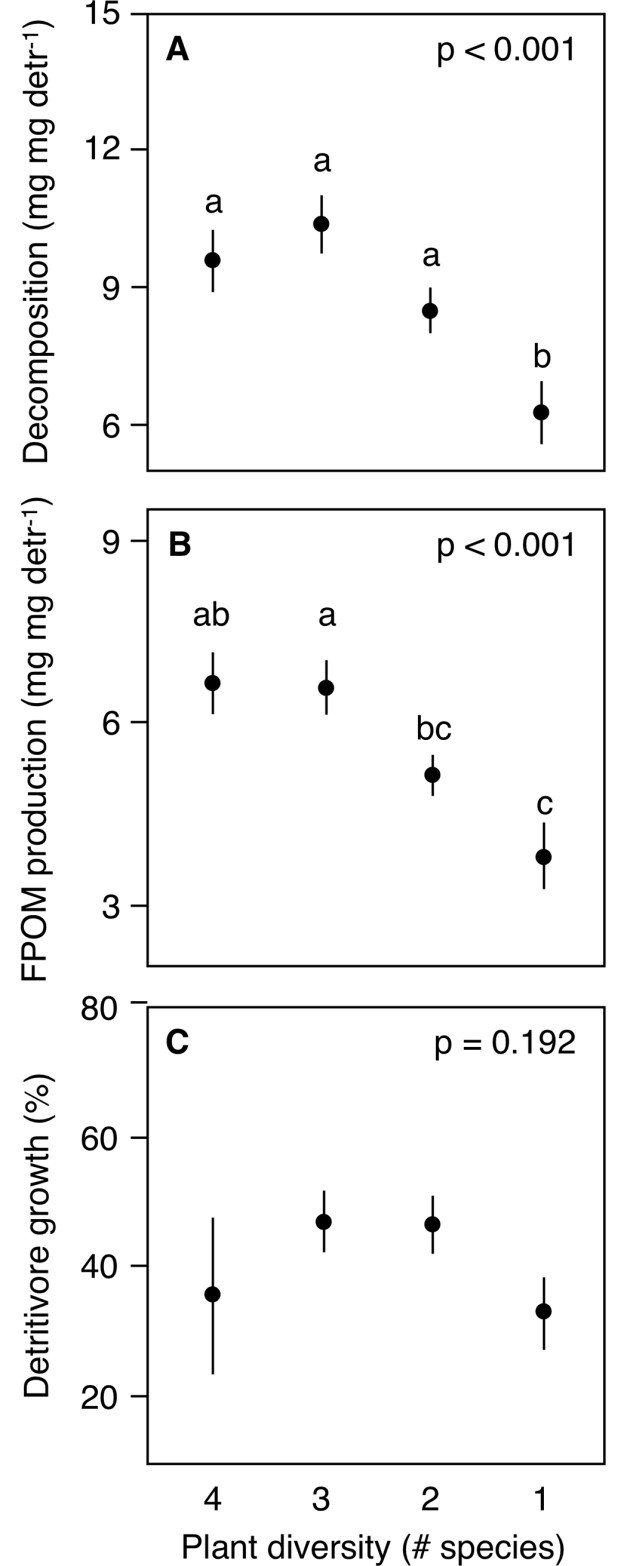
Changes in mean (± SE) (A) litter decomposition (mg leaf mg detritivore^-1^), (B) FPOM production (mg FPOM mg detritivore^-1^) and (C) detritivore growth (%) with plant diversity loss from 4 to 1 species, in microcosms with detritivores. Different lower-case letters represent significant differences across treatments (*p* < 0.05).

**Fig 3:**
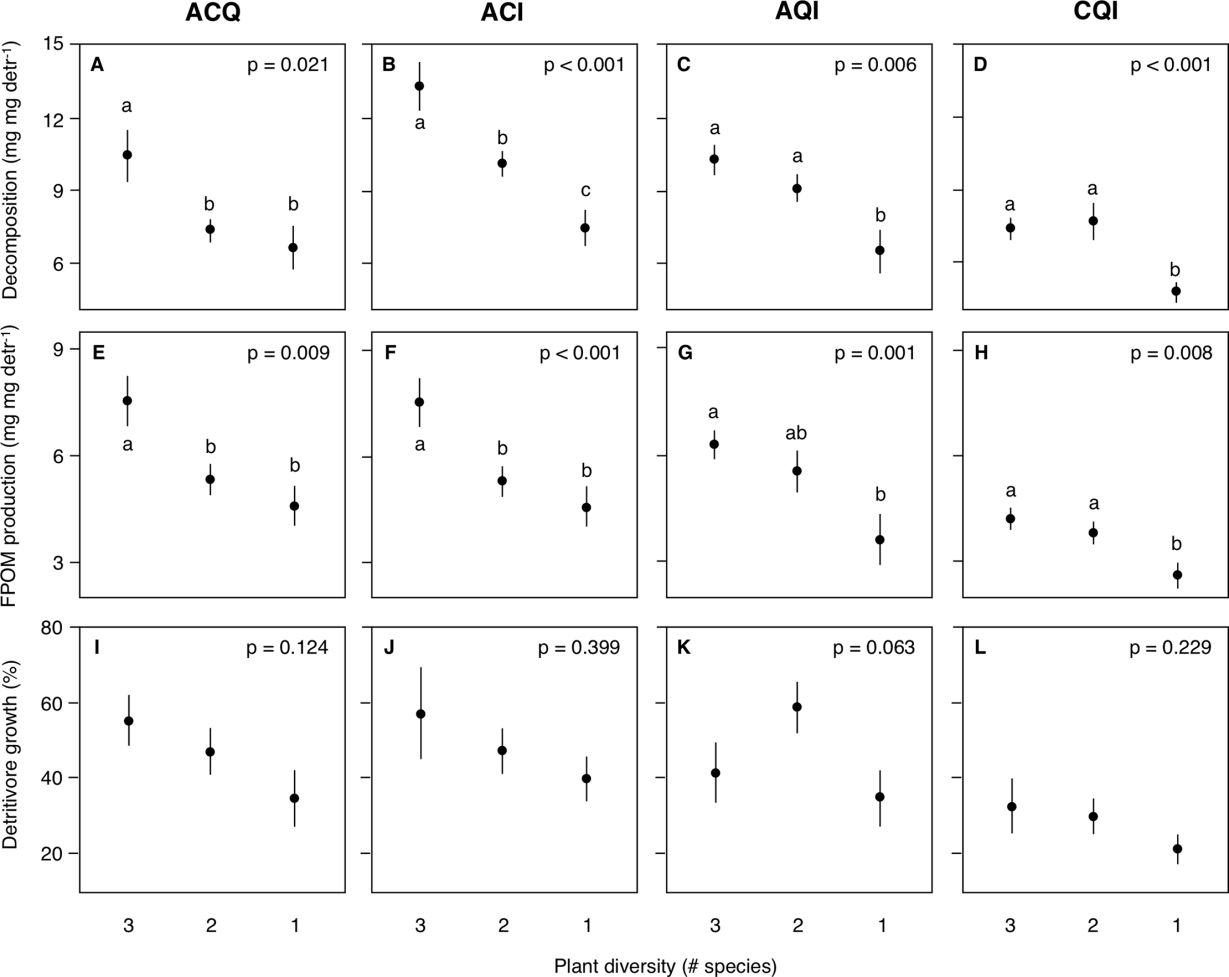
Changes in mean (± SE) (A-D) litter decomposition (mg leaf mg detritivore^-1^), (E-H) FPOM production (mg FPOM mg detritivore^-1^) and (I-L) detritivore growth (%) with plant diversity loss from 3 to 1 species in the different 3-species litter mixtures. A: *Alnus glutinosa*; C: *Corylus avellana*; Q: *Quercus robur*; I: *Ilex aquifolium*), in microcosms with detritivores. Different lower-case letters represent significant differences across treatments (*p* < 0.05).

Plant diversity loss from 4 to 1 species in ACQI reduced decomposition and FPOM production in microcosms with detritivores, but not in microcosms without detritivores, and there was no effect on detritivore growth (Table 2, S1 Table; Fig 2). When 3-species litter mixtures were examined separately, the negative effect of diversity loss from 3 to 1 species on decomposition and FPOM production was significant in all mixtures; diversity loss had no effect on detritivore growth, or on any process in microcosms without detritivores (Table 2, S1 Table, Fig 3).

**Table 2:** Results of linear models exploring effects of plant diversity loss (from 4 to 1 species in ACQI, or from 3 to 1 species in ACQ, ACI, AQI and CQI) on litter decomposition (mg mg detritivore^-1^), FPOM production (mg mg detritivore^-1^) and detritivore growth (percentage) for different litter mixtures in microcosms with detritivores.

Litter mixture	df	F	p
**Litter decomposition**			
ACQI	3	6.95	< 0.001
ACQ	2	4.42	0.021
ACI	2	11.97	< 0.001
AQI	2	5.98	0.006
CQI	2	10.94	< 0.001
**FPOM production**			
ACQI	3	7.55	< 0.001
ACQ	2	5.48	0.009
ACI	2	9.12	< 0.001
AQI	2	5.37	0.030
CQI	2	5.59	0.008
**Detritivore growth**			
ACQI	3	1.62	0.192
ACQ	2	2.23	0.124
ACI	2	0.945	0.399
AQI	2	3.02	0.063
CQI	2	1.545	0.229

(df = degrees of freedom; F = F-statistic; p = p-value). A: *Alnus glutinosa*; C: *Corylus avellana*; Q: *Quercus robur*; I: *Ilex aquifolium*)

**Fig 4:**
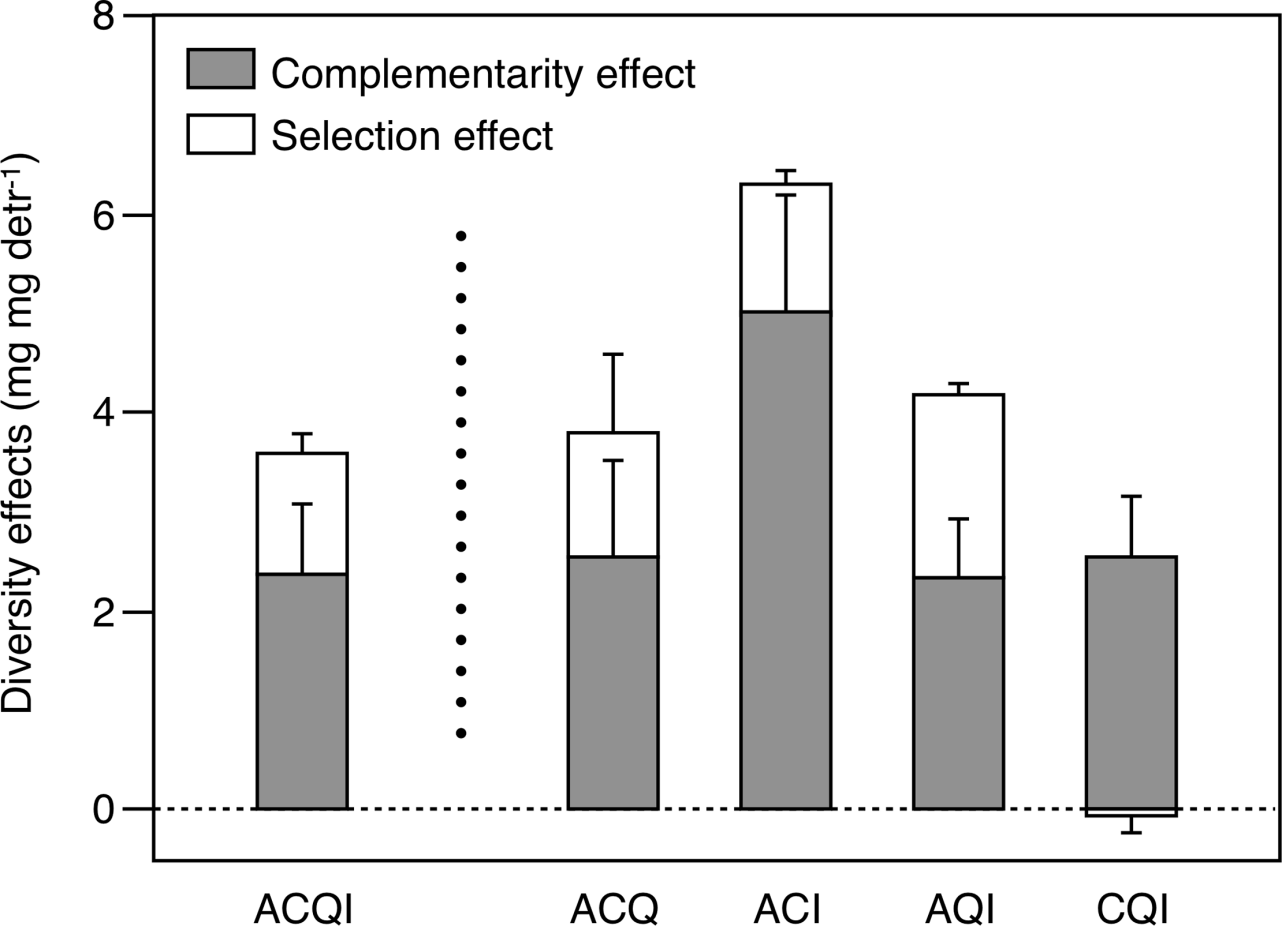
Mean (± SE) complementarity and selection effects on litter decomposition for different litter mixtures. A: *Alnus glutinosa*; C: *Corylus avellana*; Q: *Quercus robut*; I: *Ilex aquifolium*. Whole bars represent the net diversity effect (i.e., the sum of complementarity and selection effects), except for the CQI mixture where the selection effect is negative.

**Fig 5:**
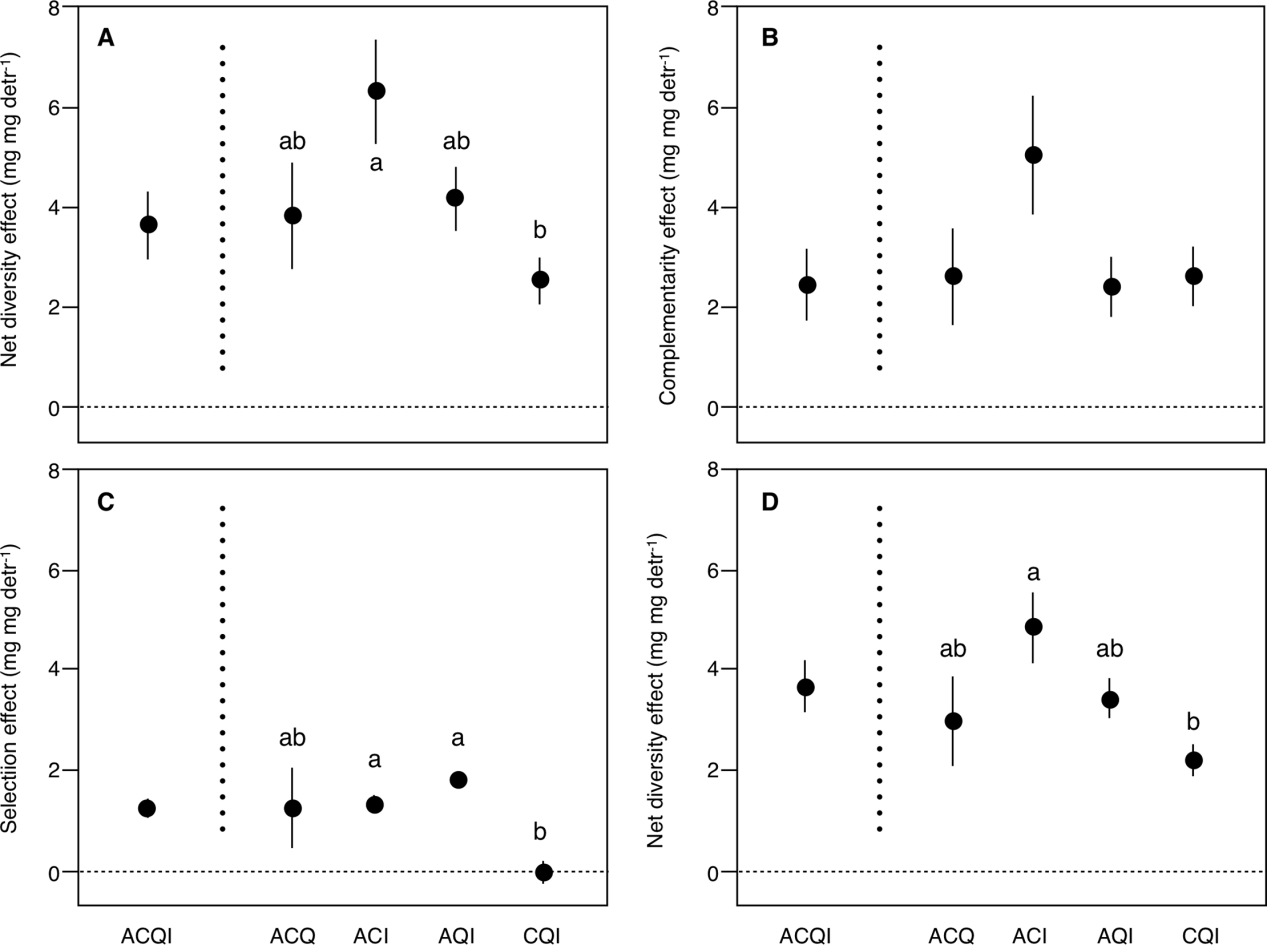
Mean (± SE) (A) net diversity, (B) complementarity and (C) selection effects on litter decomposition and (D) net diversity effects on FPOM production for different litter mixtures in microcosms with detritivores. A: *Alnus glutinosa*; C: *Corylus avellana*; Q: *Quercus robut*; I: *Ilex aquifolium*. Different lower-case letters represent significant differences across treatments (*p* < 0.05).

Net diversity effects on litter decomposition in the 4-species litter mixture were due to a combination of complementarity and selection effects, which contributed on average 66% and 34%, respectively (S2 Table, Fig 4). When 3-species litter mixtures were examined separately, the relative contribution of complementarity and selection effects was more balanced in AQI (56% and 44%, respectively), while complementarity effects had considerably higher contributions than selection effects in the other mixtures, ranging from 67% in ACQ to 99% in CQI (Fig 4). The net diversity effect on decomposition varied across litter mixtures, being significantly higher in ACI than in CQI; the complementarity effect did not vary across litter mixtures; the selecion effect was lower (and negative) in CQI than in ACI and AQI; and the net diversity effect on FPOM production was higher in ACI than in CQI (Fig 5).

The net diversity effect on decomposition was positively related to the initial average N and P concentration and leaf trait variability in litter mixtures (Table 3); thus, the highest effect occurred in the ACI mixture (Fig 5A), which had the highest P concentration and trait variability and the second highest N concentration and SLA (Table 1), and the lowest effect occurred in CQI (Fig 5A), which had the lowest N and P concentrations and trait variability and the highest toughness (Table 1).The complementarity effect on decomposition was not related to any trait or their variability (Table 3). The selection effect on decomposition was related to N and P concentration and trait variability (Table 3), with the highest effect occurring in AQI and the lowest effect in CQI (Fig 5C). The net diversity effect on FPOM production was related to P concentration and trait variability (Table 3), with the highest effect occurring in ACI and the lowest effect in CQI (Fig 5D).

**Table 3:** Results of linear models exploring the relationship between diversity effects (i.e., net diversity, complementarity or selection effect on decomposition and net diversity effect on FPOM production) and initial average leaf traits (N and P concentrations, SLA, leaf toughness and ash concentration) or trait variability in 3-species litter mixtures, in microcosms with detritivores.

Variable	F	p
**Net diversity effect on litter decomposition**		
N	5.02	0.039
P	10.225	0.005
SLA	0.06	0.800
Toughness	0.025	0.875
Ash	0.09	0.760
Trait variability	10.10	0.005
**Complementarity effect on litter decomposition**		
N	0.14	0.707
P	1.85	0.191
SLA	0.12	0.730
Toughness	0.56	0.463
Ash	0.12	0.737
Trait variability	1.80	0.197
**Selection effect on litter decomposition**		
N	8.02	0.012
P	4.70	0.044
SLA	0.02	0.888
Toughness	0.51	0.483
Ash	0.59	0.450
Trait variability	4.97	0.039
**Net diversity effect on FPOM production**		
N	3.75	0.069
P	11.39	0.004
SLA	0.425	0.523
Toughness	0.27	0.606
Ash	0.005	0.945
Trait variability	11.99	0.003

(F = F-statistic; p = p-value)

## Discussion

The results of our experiment showed that the loss of plant diversity negatively affected litter decomposition in all the litter mixtures examined. This is in agreement with a synthesis of 39 stream studies [11], but contrasts with several individual studies reporting negative or no effects of increasing plant diversity on decomposition (e.g., [21, 22, 23]). It is noteworthy that most studies examining litter diversity effects on decomposition have been conducted in streams, in contrast with experiments testing for effects of fungal or detritivore diversity, which have been mostly performed in microcosms (e.g., [24, 25, 26]) and have generally found stronger diversity effects on decomposition [27]. Field experiments have greater realism than microcosm experiments, but environmental variation often constrains the capacity for disentangling diversity-decomposition relationships, which are context-dependent [28, 29]. The choice of plant species may also have influenced the results of different studies: by selecting species belonging to different functional groups, our study and others [12] could have maximized the potential for observing effects of diversity loss on decomposition.

In our experiment, the plant diversity effect on decomposition was mediated by detritivores. Others have reported effects of plant diversity on decomposition mediated by microorganisms, but effects were more than 10 times stronger when detritivores were present [13]. Importantly, the effect of plant diversity on decomposition was highest in those mixtures with higher leaf quality and a higher variety of leaf traits, supporting our first hypothesis. Thus, the highest diversity effect on decomposition occurred in the mixture with the highest leaf quality and trait variability, and the lowest effect occurred in the mixture with the lowest leaf quality and trait variability. This result is noteworthy because previous studies had found weak or no evidence of the importance of leaf trait variability in mediating litter diversity effects on decomposition [30, 31].

Plant diversity loss affected decomposition through a combination of complementarity and selection effects, but the relative contribution of both mechanisms varied, as predicted by our second hypothesis. A positive complementarity effect was dominant in most cases (56–99% of net diversity effects), which indicated the prevalence of resource partitioning or facilitation [20]. Thus, leaves from different species may provide complementary resoruces for detritivores (e.g., different nutrients), or the presence of some species may facilitate the use of other species by detritivores. For example, Handa et al.’s [12] findings suggested the existence of nutrient transfer among species through fungal decomposers: N seemed to be transferred from leaves of N-fixer plants to leaves of non-fixers. Others have found that the presence of refractory leaves can enhance the decomposition of labile leaves [32], possibly due to a reduction in negative density-dependent effects resulting from the aggregation of detritivores in higher-quality leaves [9].

Nevertheless, a positive selection effect was also important in our study (21%-44% of net diversity effects), except for the CQI mixture (the only mixture not containing *Alnus*), where the selection effect was negligible. Thus, as expected, a selection effect only appeared in the initial presence of *Alnus*, which is consistently preferred by detritivores over other species in experiments (e.g., [13, 14, 33, 34]). *Alnus* leaves are very rich in N and P and highly palatable, so the loss of this species from litter mixtures seems to have a large effect on decomposition. Others have also shown that diversity effects on decomposition can depend on whether the remaining species are more or less preferred by detritivores [35], but our study gives further light on the mechanisms underlying such effects. Thus, when *Alnus* leaves are present in a mixture, this species comes to dominate the decomposition process, even if present in equal abundances to other species; this differs from primary productivity, where the selection effect is often associated with numerical abundance of the dominant species [20].

We found that plant diversity loss negatively affected FPOM production, so our third hypothesis was partly supported. Again, the effect of plant diversity on FPOM production was mediated by detritivores, as there was no effect in microcosms without detritivores. FPOM production is tightly linked to detritivore-mediated litter decomposition, as FPOM is composed of their faeces [36] and small litter fragments produced as a result of their feeding activity [4]. Another experiment also found that FPOM production was reduced when 3-species litter mixtures lost one or two species [37]. Here we further showed that the effect of plant diversity on FPOM production occurred across different litter mixtures but its magnitude varied, being stronger in ACI and weaker in CQI. This variation was again related to P concentration and trait variability, both of which were highest in ACI and lowest in CQI.

Surprisingly, even if plant diversity effects on decomposition and FPOM production were mediated by detritivores, detritivore growth was not affected. This lack of effect could be to the fact that all litter mixtures might have offered sufficient resources for maximum growth [13, 38]. However, there was a trend for detritivore growth to decrease with plant diversity loss in most mixtures (ACQ, ACI and CQI), even if the trend was not significant; this suggests that detritivore growth responds similarly to decomposition and FPOM production, but we may have not been able to detect a significant effect because of the higher data variability. Such variability could be due to the fact that detritivore initial biomass was not measured directly, but rather estimated from caddisfly case length; or to the relatively short experimental time, although this is unlikely because mean growth was 42% during the experiment. Another plausible explanation for the lack of effect of diversity on growth is that detritivores can modulate their growth efficiency [39]–that is, change their assimilation and eggestion efficiencies in order to balance their stoichiometric demands [40]. For example, detritivores may be able to modify the composition of their fecal pelets in order to maintain their body composition regardless of their diet [41], or reduce their carbon use eficiency when resources are nutrient limited [42].

Our results support the existence of widespread effects of riparian plant species loss on key stream ecosystem processes driving detrital food webs, such as litter decomposition and FPOM production, both of which are slowed as a result of species loss. This is in agreement with a synthesis reporting a negative effect of plant diversity loss on litter decomposition [11] and with the only available study reporting a negative effect of plant diversity loss on FPOM production to our knowledge [37]. However, our results further suggest that plant diversity effects on these processes can be stronger or weaker depending on which riparian species are present originally in litter mixtures. Thus, litter mixtures that initially are of higher quality (i.e., with higher N and/or P concentrations) are strongly affected by plant diversity loss, as are litter mixtures with higher varaibility of leaf traits. A key outcome of our experiment is that the risk of species loss to stream ecosystem functioning was largely due to a loss of complementarity, but selection effects were also important in mixtures containing *Alnus*. This highlights the importance of riparian species such as *Alnus glutinosa*, which provide litter of high quality, their loss being likely to have substantial detrimental effects on stream ecosystem functioning, particularly when other riparian species are of lower quality.

## Supporting information

S1 Data Excel spreadsheet containing the underlying numerical data for all figures and tables. (XLSX)

S1 Table Effects of plant diversity loss on litter decomposition (mg) and FPOM production (mg) for the 4-species litter mixture (ACQI) and the different 3-species mixtures (ACQ, ACI, AQI and CQI) in microcosms without detritivores, examined with linear models. A: *Alnus glutinosa*; C: *Corylus avellana*; Q: *Quercus robur*; I: *Ilex aquifolium*.
(DOCX)

S2 Table Mean (± SE) net diversity, complementarity and selection effects on litter decomposition, and net diversity effect on FPOM production, for the 4-species litter mixture (ACQI) and the different 3-species mixtures (ACQ, ACI, AQI and CQI) in microcosms with detritivores. A: *Alnus glutinosa*; C: *Corylus avellana*; Q: *Quercus robur*; I: *Ilex aquifolium*
(DOCX)
