# Supplementary material for: Leaf traits drive plant diversity effects on litter decomposition and FPOM production in streams
Source: PLoS One. 2018 May 29;13(5):e0198243. doi: 10.1371/journal.pone.0198243 (PMC5973617; doi:10.1371/journal.pone.0198243)
Supplement: S1 Table — A: Alnus glutinosa; C: Corylus avellana; Q: Quercus robur; I: Ilex aquifolium. (DOCX) [file pone.0198243.s002.docx]

**Table S1.** Effects of plant diversity loss on litter decomposition (mg) and FPOM production (mg) for the 4-species litter mixture (ACQI) and the different 3-species mixtures (ACQ, ACI, AQI and CQI) in microcosms without detritivores, examined with linear models.

| Mixture | df | MS | F | p |
| --- | --- | --- | --- | --- |
| Litter decomposition |  |  |  |  |
| ACQI | 3 | 26.88 | 0.06 | 0.978 |
| ACQ | 2 | 8.51 | 0.17 | 0.843 |
| ACI | 2 | 14.82 | 0.02 | 0.977 |
| AQI | 2 | 60.22 | 0.11 | 0.895 |
| CQI | 2 | 10.89 | 0.02 | 0.984 |
| FPOM production |  |  |  |  |
| ACQI | 3 | 1.25 | 1.27 | 0.291 |
| ACQ | 2 | 0.67 | 0.51 | 0.606 |
| ACI | 2 | 1.29 | 1.05 | 0.363 |
| AQI | 2 | 0.50 | 0.67 | 0.518 |
| CQI | 2 | 0.64 | 0.54 | 0.586 |

(A: *Alnus glutinosa*; C: *Corylus avellana*; Q: *Quercus robur*; I: *Ilex aquifolium*)
