# Supplementary material for: Leaf traits drive plant diversity effects on litter decomposition and FPOM production in streams
Source: PLoS One. 2018 May 29;13(5):e0198243. doi: 10.1371/journal.pone.0198243 (PMC5973617; doi:10.1371/journal.pone.0198243)
Supplement: S2 Table — A: Alnus glutinosa; C: Corylus avellana; Q: Quercus robur; I: Ilex aquifolium (DOCX) [file pone.0198243.s003.docx]

**Table S2**. Mean (±SE) net diversity, complementarity and selection effects on litter decomposition, and net diversity effect on FPOM production, for the 4-species litter mixture (ACQI) and the different 3-species mixtures (ACQ, ACI, AQI and CQI) in microcosms with detritivores .

|  | Net diversity effect | Complementarity effect | Selection effect |
| --- | --- | --- | --- |
| Litter decomposition |  |  |  |
| ACQI | 3.64 ± 0.68 | 2.39 ± 0.73 | 1.25 ± 0.20 |
| ACQ | 3.81 ± 1.06 | 2.57 ± 0.96 | 1.25 ± 0.78 |
| ACI | 6.31 ± 1.05 | 5.01 ± 1.19 | 1.30 ± 0.18 |
| AQI | 4.16 ± 0.64 | 2.34 ± 0.59 | 1.82 ± 0.13 |
| CQI | 2.53 ± 0.48 | 2.56 ± 0.61 | -0.03 ± 0.21 |
| FPOM production |  |  |  |
| ACQI | 3.62 ± 0.51 |  |  |
| ACQ | 2.92 ± 0.89 |  |  |
| ACI | 4.81 ± 0.71 |  |  |
| AQI | 3.36 ± 0.39 |  |  |
| CQI | 2.13 ± 0.30 |  |  |

( A: *Alnus glutinosa*; C: *Corylus avellana*; Q: *Quercus robur*; I: *Ilex aquifolium*)
